# BioGraphE: high-performance bionetwork analysis using the Biological Graph Environment

**DOI:** 10.1186/1471-2105-9-S6-S6

**Published:** 2008-05-28

**Authors:** George Chin, Daniel G Chavarria, Grant C Nakamura, Heidi J Sofia

**Affiliations:** 1 High Performance Computing Group, Computational Sciences and Mathematics Division, Pacific Northwest National Laboratory, P.O. Box 999, Richland, Washington, USA; 2 Information Analytics Department, Computational and Statistical Analytics Division, Pacific Northwest National Laboratory, P.O. Box 999, Richland, Washington, USA; 3 Computational Biology and Bioinformatics Department, Computational Sciences and Mathematics Division, Pacific Northwest National Laboratory, P.O. Box 999, Richland, Washington, USA

## Abstract

**Background:**

Graphs and networks are common analysis representations for biological systems. Many traditional graph algorithms such as k-clique, k-coloring, and subgraph matching have great potential as analysis techniques for newly available data in biology. Yet, as the amount of genomic and bionetwork information rapidly grows, scientists need advanced new computational strategies and tools for dealing with the complexities of the bionetwork analysis and the volume of the data.

**Results:**

We introduce a computational framework for graph analysis called the Biological Graph Environment (BioGraphE), which provides a general, scalable integration platform for connecting graph problems in biology to optimized computational solvers and high-performance systems. This framework enables biology researchers and computational scientists to identify and deploy network analysis applications and to easily connect them to efficient and powerful computational software and hardware that are specifically designed and tuned to solve complex graph problems. In our particular application of BioGraphE to support network analysis in genome biology, we investigate the use of a Boolean satisfiability solver known as Survey Propagation as a core computational solver executing on standard high-performance parallel systems, as well as multi-threaded architectures.

**Conclusion:**

In our application of BioGraphE to conduct bionetwork analysis of homology networks, we found that BioGraphE and a custom, parallel implementation of the Survey Propagation SAT solver were capable of solving very large bionetwork problems at high rates of execution on different high-performance computing platforms.

## Background

Networks and graphs are well-studied constructs in computer science and applied mathematics. As the sizes and complexities of networks grow, however, critical limitations arise that restrict a scientist's ability to analyze a large network, both computationally and cognitively. From our observations, humans appear to be capable of comprehending networks that contain up to about 100 nodes. Single-processor computers, on the other hand, can often process graphs of up to 100,000s of nodes, depending on the algorithm. Yet, in many scientific fields, networks often arise that may consist of millions to hundreds of millions of nodes. In a world of continually growing scientific data and information spaces, scientists need advanced new strategies, tools, and computer systems to effectively and efficiently process and analyze large-scale scientific networks.

Data complexity and volume issues have recently become especially significant in biology as the result of high-throughput data production in genomics and proteomics. Networks are a fundamental concept in biological systems, and their analysis is an essential capability. Of great interest are methods for discovering bionetworks from diverse types of data, storing, representing, comparing, and extracting features from them, and computing upon dynamic bionetworks. Yet, despite these trends, it is also increasingly common to hear biologists use the term "giant hairball" to convey their experiences working with large, complex bionetworks in research.

We believe that unlocking the real potential of these networks for biologists will require a significant investment in high-performance computing (HPC) and innovative computational systems and techniques. These approaches have been applied less often in biology, yet biological systems have many intriguing and ideal properties for driving the development of advanced computational strategies and high-powered solutions. Biological computing involves critical issues in data-intensive computing, defined as "applications to explore, query, analyze, visualize, and in general process very large-scale datasets" [1]. In this context, scale relates to both absolute data size and the algorithms and applications that can process large-scale data.

In this paper, we introduce the Biological Graph Environment (BioGraphE), which offers novel graph approaches, techniques, and tools in an integrated framework to address data-intensive computing challenges associated with large-scale bionetworks. BioGraphE will enable biology researchers and computational scientists to identify and deploy network analysis applications and to easily connect them to efficient and powerful computational software and hardware that are specifically designed and tuned to solve complex graph problems.

## Results

### Graph problems in genome biology

Many areas of biological research are poised to benefit from advances in network analysis. Sources of biological networks include protein-protein interactions, gene expression from microarrays, metabolic pathways, and signal transduction cascades. We have selected genome biology problems in this project for several reasons. These results are important to biologists in the study of how proteins function in the cellular machinery. We are able to build networks in genome biology in a highly tunable fashion: they can be made as large or small, as simple or complex as needed, in large numbers, and with correct biological encodings. The data is freely available in public databases with large quantities of nodes for these networks as well as diverse types of interconnections. Edges based on homology can be defined in high-throughput using the BLAST [2] algorithm and these comparisons can be performed very efficiently with the ScalaBLAST implementation [3]. Other types of edges may also be retrieved from the databases. Finally, we are able to extract right or wrong answers from these networks to provide useful feedback on the analysis.

**Figure 1:**
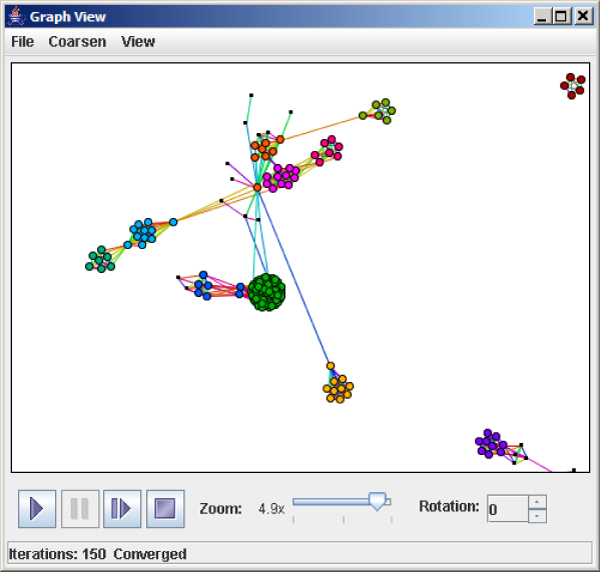
**K-cliques in homology networks**. Large cliques identify families of proteins in a homology network. Cliques representing different protein subtypes are shown in the graph in different colors.

We have produced a series of protein homology networks for testing BioGraphE based on sigma 70 signaling pathways. Sigma 70 regulatory proteins are of interest for bioenergy applications. Sigma and anti-sigma factor proteins are modular protein pairs that function as on/off switches in many microbial signaling pathways. Recently, we have shown that seemingly discrete clusters of anti-sigma factors are linked together by a previously unrecognized protein domain [4]. In a protein homology network this result can be detected as bridge proteins that link densely clustered regions of anti-sigma factor homology networks. We illustrate the use of several common graph algorithms with protein homology networks, including k-clique, subgraph matching, and k-coloring. Protein families that are functional subtypes are often fully connected in a homology network, and thus, appear as cliques. Figure 1 shows a Similarity Box [5] visualization of the maximum cliques found from a bionetwork derived from chromosomal neighbors of sigma 70 proteins.

**Figure 2:**
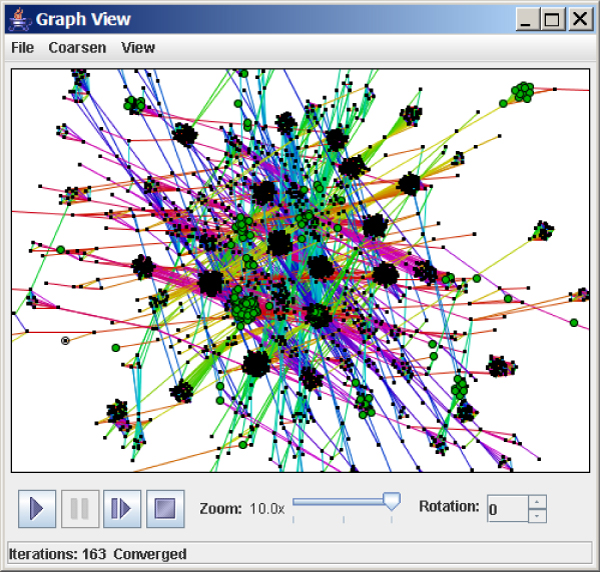
**Subgraph pattern matching in homology networks**. Subgraph pattern matching may be applied to search for sets or classes of proteins within homology networks. Nodes of the identified subgraph are plotted in green. In this example, the identified proteins have a particular chromosome orientation and fall within specific clusters representing different protein families.

Subgraph pattern matching is illustrated in Figure 2. The subgraph highlighted in green consists of gene products with a particular orientation in the chromosome near sigma 70 factors. The highlighted proteins are not randomly placed throughout the network, but rather fall within specific clusters that represent different protein families.

**Figure 3:**
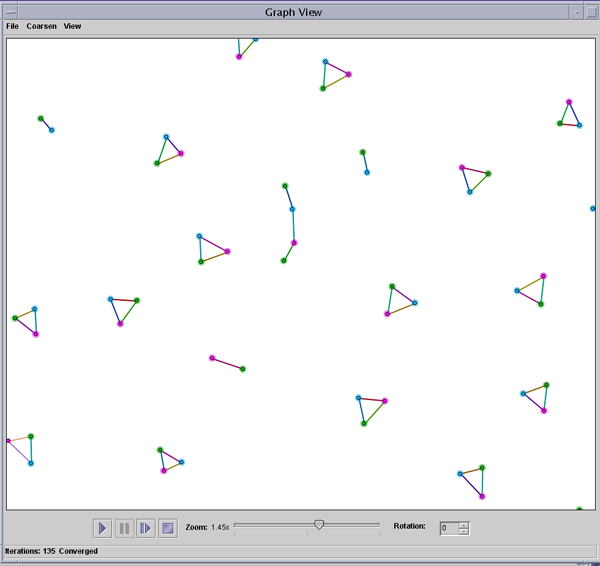
**K-coloring of homology networks**. K-coloring may be used to study bionetworks generated from bidirectional best hits. The color of a protein specifies the strain to which that protein belongs. The network is designed such that adjacent nodes will never be of the same color.

K-coloring is illustrated in Figure 3. The patterns in this graph identify subtle evolutionary relationships between proteins from three strains of *Rhodobacter sphaeroides*, each marked in a different color. The 3-cliques display orthologous proteins based on bidirectional best hits, while proteins in the open subgraphs are homologs but not likely to be orthologs.

### Computational framework for large-scale graph analysis

Graph algorithms are known to be computationally expensive for large graphs because they often involve comparisons of each node to every other node in the graph. As the number of nodes increase, the number of comparisons will often increase exponentially. In fact, many general graph problems such as k-clique, Hamiltonian paths and cycles, and subgraph isomorphism are NP-complete (i.e. there is no known polynomial time algorithm).

To facilitate complex, large-scale graph analysis, we seek a general solution and computational framework to enable scientists to deploy and integrate new graph algorithms and techniques as needed. Rather than continuously building customized, one-off graph solutions, we wish to develop and provide an extensible and uniform analysis platform built on modern computational approaches and systems.

**Figure 4:**
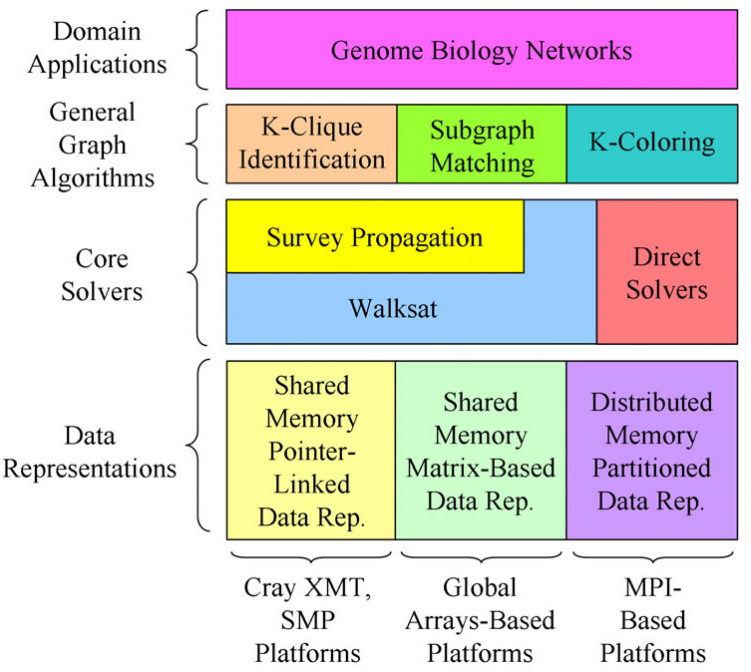
**The computational framework of BioGraphE**. Different graph problems are applied in network analysis and may be reduced to a form for input into general solvers or directly solved on underlying high-performance systems using specific data representations.

We introduce *BioGraphE *as a high-performance computational framework for the analysis of complex networks from biology and potentially other domains as well. As shown in Figure 4, BioGraphE is designed to integrate a variety of graph analysis applications into a common suite of tools. Our approach is to identify relevant graph problems and reduce them into equivalent problems that may be addressed by efficient solver implementations. Examples of potential general solvers are ones built for Boolean satisfiability equations and integer linear programming. As an alternative to using a general solver, graph problems may also be directly solved by implementing specific solutions on high-performance systems.

To achieve the best performance, direct and indirect graph solvers should be implemented on high-performance computing (HPC) architectures such as compute clusters, shared-memory SMP and NUMA platforms, as well as multithreaded architectures such as the Sun Niagara and Cray MTA-2. To best utilize the features and capabilities of individual HPC platforms, computational solvers should utilize data structures and synchronization code that are most efficient for the individual platforms.

As a computational framework for bionetwork analysis, BioGraphE has distinct benefits including:

• The BioGraphE framework is extensible and allows core solvers to be added and new graph algorithms to be built on top of the algorithmic toolkit.

• Core solvers should address well-studied problems in computer science, where efficient heuristics and algorithms are available. Specific domain problems and applications, on the other hand, have had much less scrutiny and evolution, and thus, are much less likely to garner optimized solutions.

• Core solvers should provide a very expressive language or interface to which many different kinds of graph problems may be translated.

• Once the core solvers have been ported and optimized to run on a HPC machine, domain problems that are passed to the solvers should automatically gain the performance and benefits of the HPC machine without requiring recoding to that platform.

### Bionetwork analysis

When applying BioGraphE to support bionetwork analysis, we had to make some choices regarding the software and hardware to be utilized. We sought a core solver that would be capable of efficiently solving NP-complete problems, since many of the graph algorithms we wished to apply were also NP-complete. We also sought a general solver that would accept a wide range of problems, had known formulations for graph problems, and had strong potential for code parallelization. For these reasons, we selected a modern solver for Boolean satisfiability (SAT) equations called Survey Propagation (SP) [6,7] as a core solver.

SAT is a well-known NP-complete constraint-satisfaction problem. Various graph problems such as k-clique, k-coloring, and Hamiltonian paths and cycles may be reduced to SAT equations using known efficient reduction algorithms [8]. SP is a modern SAT solver originally developed to study the physics of complex systems such as spin glasses. It has proven to be successful on many large satisfiability problems beyond the reach of other SAT methods.

#### SAT solving using Survey Propagation

Given randomly generated formulas of M clauses of exactly K literals over N Boolean variables, research has shown that hard instances of random K-SAT exist when the formulas are near specific threshold values of α = M/N. K-SAT problems moderately below α are under-constrained and easily satisfiable, while those moderately above α are over-constrained and generally unsatisfiable. K-SAT problems near α identify a critical phase transition region where solutions are difficult and computationally expensive to obtain. In the case of random 3-SAT, the α threshold value was found to be approximately 4.2 [7]. More specifically, α values between 3.921 and 4.267 were considered to represent the hard region for random 3-SAT.

SP was designed to solve SAT problems that fall within the hard region. To initially evaluate SP, we executed the algorithm on a personal computer running Window XP on a single Intel 2.00 GHz Pentium M processor. We tested randomly-generated 3-SAT formulas consisting of various numbers of variables and the number of clauses equal to 4.2 times the number of variables. Thus, the 3-SAT problems fell within the hard region. As shown in Table 1, the SP + *Walksat *combination had increasingly better performance than executing *Walksat *alone for 3-SAT problems consisting of 2500 variables and above. For every test, SP computed a partial solution with a subset of variables defined and clauses satisfied. With our test cases, partial solutions passed to *Walksat *always had α values much lower than the critical range of 3.921 and 4.267, and thus, were easily satisfiable.

**Table 1:** Sequential SAT solver statistics and execution times on random 3-SAT equations. Initial statistics and wall clock times for baseline testing of the Mézard and Zecchina sequential implementation of Survey Propagation and Walksat SAT solvers on a personal computer running Windows XP on a single Intel 2.00 GHz Pentium M processor.

**SP Variables**	**SP Clauses**	**α**	**Walksat Variables**	**Walksat Clauses**	**Walksat α**	**Walksat Exec Time (alone)**	**SP Exec Time (comb.)**	**Walksat Exec Time (comb.)**	**SP+Walksat Exec Time (comb.)**
1,000	4,200	4.20	790	2,595	3.28	0.05	0.34	0.02	0.36
2,500	10,500	4.20	429	485	1.13	3.83	2.78	0.02	2.80
5,000	21,000	4.20	2,785	6,939	2.49	22.41	3.30	0.02	3.31
7,500	31,500	4.20	4,633	12,569	2.71	457.95	5.25	0.34	5.59
10,000	42,000	4.20	5,464	13,465	2.46	492.75	9.30	0.05	9.34
50,000	210,000	4.20	26,318	63,467	2.41	3,388.52	98.61	1.64	100.25
100,000	420,000	4.20	56,649	143,932	2.54	5,121.84	231.27	4.19	235.45

A number of issues should be noted when using SP to solve hard random K-SAT problems. First, SP is a heuristic algorithm that has no guarantee of convergence, but still has been found to converge for many hard K-SAT problems that other K-SAT implementations were unable to solve. Furthermore, SP has been found to be particularly effective in determining the satisfiability of K-SAT formulas, but has some difficulty determining unsatisfiability [7]. Finally, SP has mainly been tested for randomly-generated K-SAT formulas. Evaluations of SP's performance on nonrandom K-SAT formulas have been limited.

#### Parallel implementation of Survey Propagation

Although rapid progress has been made in SAT solvers, most are sequential and few are parallel [10], and are thus limited to the capabilities of a single workstation. In order to enable the solution of very large sets of SAT equations (such as those derived from large biologically-originated graphs), we need the parallel processing capabilities and large aggregated memory spaces of HPC systems.

Given the advantages exhibited by SP over traditional SAT solvers for very large sets of equations, we implemented a parallel version of SP. SP operates by repeatedly updating weights associated with the variable and clause nodes until a fixed point is reached when the difference between successive updates falls below a specific threshold. The variables must be updated in a random order that changes at each iteration step. The fine-grained nature of the synchronization in this computation makes it more suitable for a parallel implementation on a multiprocessor with a shared address space than on a network of commodity workstations.

#### Parallel computing environments

We implemented two versions of the parallel SP application:

• A portable, shared-memory OpenMP implementation.

• An experimental shared-memory, multithreaded version for the Cray MTA-2 and XMT systems [12,13]. The MTA platform has been found to be very effective for irregular and graph-based applications [14-16].

Our testing platforms were a Sun Fire T2000 server with an 8-core Sun UltraSPARC T1 ("Niagara") processor and a 40-processor Cray MTA-2 system located at the Cray facilities. More details about these platforms can be found in [14]. On the Sun Niagara server, we executed the OpenMP version compiled with the Sun compiler suite. On the MTA-2 system, we executed our custom multithreaded version.

**Figure 5:**
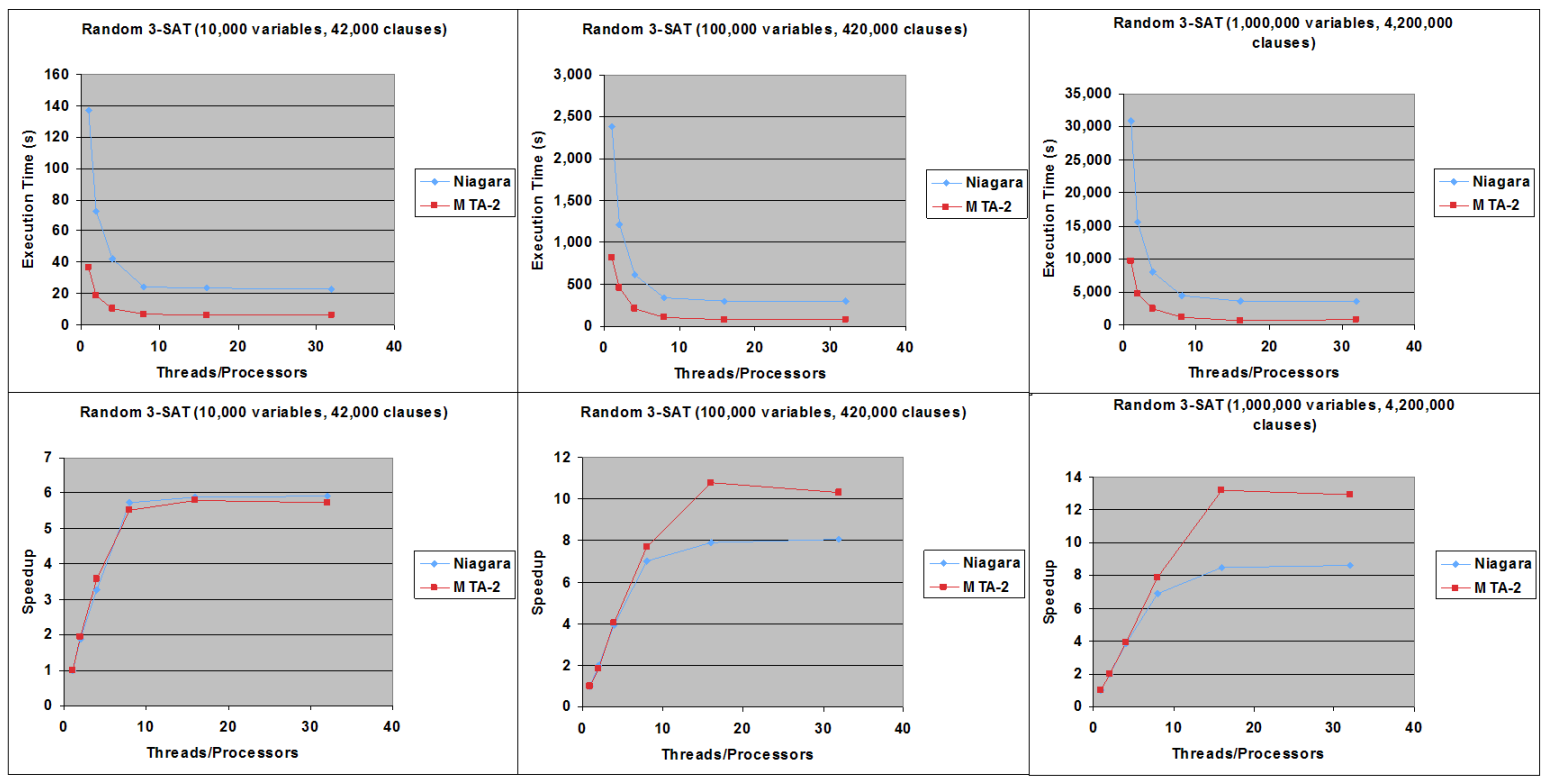
**Parallel SP solver execution statistics on random 3-SAT equations**. Initial wall clock times and speedup rates for parallel SP algorithm on an 8-core Sun Microsystems Niagara and 40-processor Cray MTA-2.

As part of our characterization of the SP algorithm, we tested both parallel implementations with a series of problems that are designed to fall in the "hard" SAT space where SP is especially known to outperform other SAT solvers. All of the cases we tested were random instances generated with α = 4.2 that places the problem within the "hard" SAT region. As shown by the actual clock times of Figure 5, the execution time of this algorithm implementation scales non-linearly with the size of the problem instance. Overall, we found the MTA-2 to have a 2.66 to 4.98 times improvement in execution times over the Niagara. The speedup charts of Figure 5 also illustrate that the parallel SP code exhibits an almost linear speedup within the same problem size when utilizing from one to eight processors on both the Niagara and the MTA-2. The efficiency generally degrades on both machines, however, when the number of processors exceeds eight except in the case of the MTA-2 running the largest SAT problem (1,000,000 variables, 4,200,000 clauses). In this one case, MTA-2 further exhibits linear speedup of up to sixteen processors. In addition, the MTA-2 was able to achieve higher overall speedup rates than the Niagara. For the largest SAT problem, for example, MTA-2 was able to achieve a speedup rate of up to 13.17, while Niagara's highest speedup rate was about 8.65.

### K-clique analysis of homology networks

As an initial, proof-of-principle problem, we chose to apply the k-clique problem on a collected set of protein homology networks, which are graphs where nodes represent proteins and edges represent similarity between proteins measured by the BLAST algorithm [2]. Correspondingly, we have built software to perform the transformation or reduction of a protein homology network specification into a set of Boolean satisfiability equations for finding a k-clique in the network. To test this software, we prepared a series of graphs that represent specific biological relationships that we understood well.

#### K-clique to SAT reduction

We implemented a k-clique to SAT reduction algorithm that takes a particular input graph and produces SAT equations. The SAT equations, in turn, will be solved by the SAT solvers in BioGraphE. A k-clique is a subgraph consisting of k nodes, all of which have edges to one another.

As shown in Table 2 and 3, we applied the k-clique to SAT reduction to a number of homology networks. In the first set of test cases, we started by looking for 3-cliques in a specific homology network. To find the maximum clique, we incrementally increased the size of the clique until no cliques in the network were found. An interesting side effect of the reduction and SP algorithm was that the set of true variables produced by SP was always associated with the same clique in the network. We used this information to more efficiently adjust the size of clique for which we were looking. For instance, if we analyzed a homology network for 3-cliques and the SP algorithm returned eight true variables, then we should know that at least one clique of size eight existed in the network. We would immediately start searching for 9-cliques in the network on our way to finding the maximum clique.

**Table 2:** Impact of clique size on k-clique to SAT reduction. Statistics from the reduction of nodes and edges from the k-clique problem to the equivalent variables and clauses of SAT equations for different values of k.

**Nodes**	**Edges**	**K**	**Vars**	**Clauses**	**α**
148	6,186	3	444	70,089	157.86
148	6,186	5	740	194,935	263.43
148	6,186	10	1,480	780,470	527.34
148	6,186	20	2,960	3,123,340	1,055.18
148	6,186	30	4,440	7,028,610	1,583.02
148	6,186	40	5,920	12,496,280	2,110.86
148	6,186	50	7,400	19,526,350	2,638.70
148	6,186	60	8,880	28,118,820	3,166.53
148	6,186	67	9,916	35,063,177	3,536.02
148	6,186	68	10,064	36,117,724	3,588.80

**Table 3:** Impact of graph size on k-clique to SAT reduction. Statistics from the reduction of nodes and edges from the k-clique graph problem to the equivalent variables and clauses of SAT equations for different graph sizes.

**Nodes**	**Edges**	**K**	**Vars**	**Clauses**	**α**
148	6,186	3	444	70,089	157.86
248	11,529	3	744	224,325	301.51
533	52,055	3	1,599	1,038,501	649.47
781	63,939	3	2,343	2,449,329	1,045.38
1,270	196,121	3	3,810	6,303,957	1,654.58
3,741	217,350	3	11,223	61,979,313	5,522.53

Table 2 also shows how the size of the SAT problem rapidly increases with clique size. Our clique-to-SAT reduction approach produces O(n^2^k^2^) clauses with O(nk) variables, where n is the number of nodes in the graph and k is the size of the clique.

In the second set of test cases, we applied SP to homology networks of different sizes. Table 3 shows that the SAT problem also rapidly increases with the overall size and complexity of the homology network.

In both the cases of increasing clique size k or the number of nodes n, a very large number of clauses are generated and the corresponding α values of the SAT equations are very high compared to the 4.2 phase transition region for 3-SAT equations. One might expect the produced SAT equations to be over-constrained and unsolvable. Nevertheless, the SAT equations from the reduction are generally solvable using SP. This interesting result requires further investigation as well as the exploration of alternative reduction strategies to generate SAT equations from k-clique problems.

We should also note that the Boolean formulas produced from the reduction are in the form of 2- and n-clauses. Since most SAT phase transition studies have focused on SAT equations with fixed clause sizes (e.g., 3-SAT), a relevant research effort would be to examine and characterize the phase transition region of our particular set of SAT equations that evolved directly from bionetworks. Part of this research should involve simplifying the SAT equations to more efficient forms.

In addition, we are evaluating three additional reduction algorithms for k-clique in the literature [8] that require a smaller number of variables for the reduction, as well as reductions for other graph methods. It is important to note that these more sophisticated reductions will not necessarily lead to faster execution speeds for the overall graph analysis procedure, since we expect the overall runtime to be dominated by the SP solver. In particular, the runtime should be dominated by how "hard" the SAT instances are as defined by the ratio of clauses to variables. For 3-SAT, it has been experimentally determined that a ratio of clauses to variables of approximately 4.2 may lead to SAT instances with exponential solution times. We are in the process of studying the behavior of our reduction-generated SAT instances and characterizing them with respect to their solution difficulty.

#### Parallel performance of Survey Propagation

**Figure 6:**
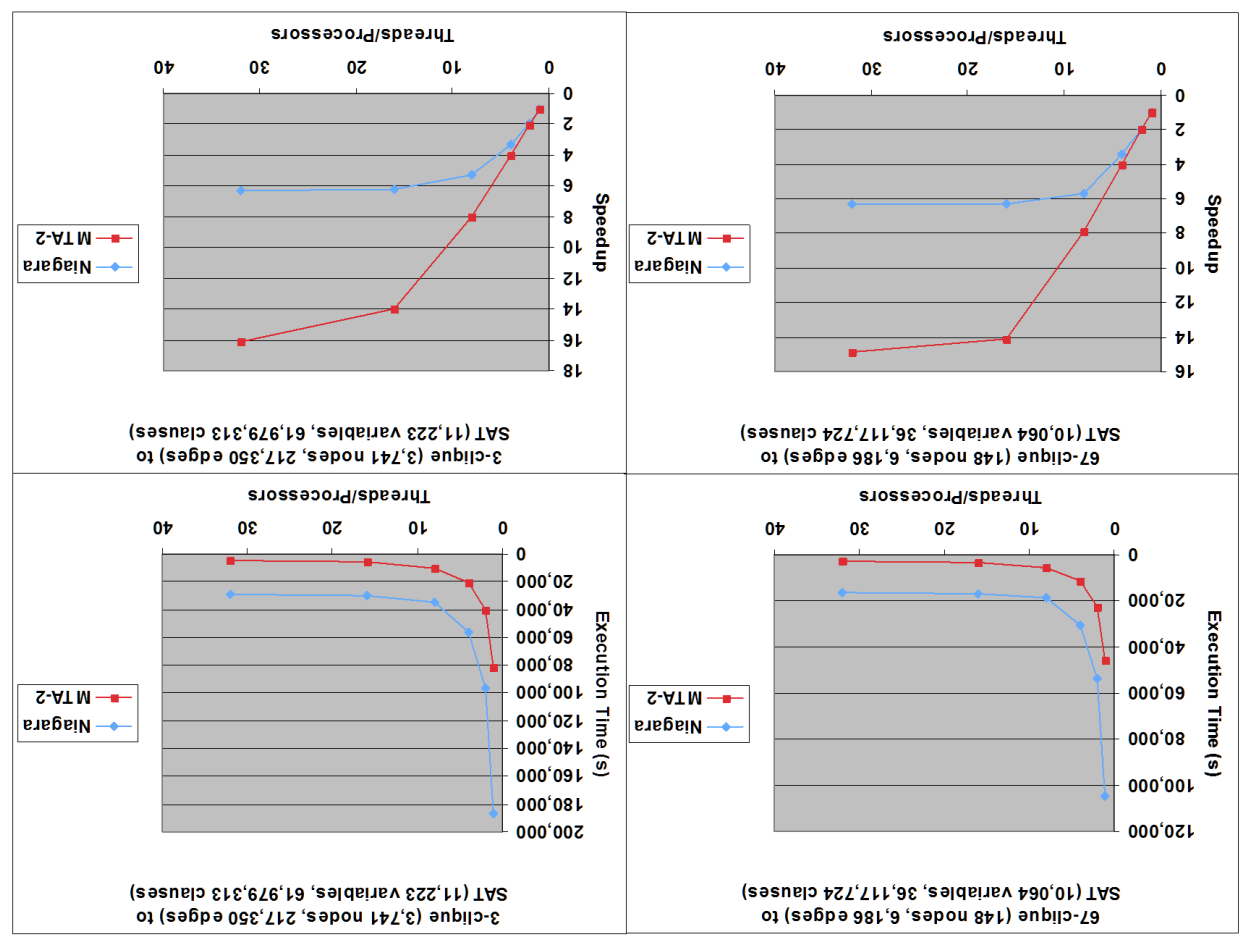
**Parallel SP solver execution statistics on SAT equations generated from genome biology graphs**. Initial wall clock times and speedup rates for parallel implementation of the Survey Propagation algorithm on an 8-core-processor Sun Microsystems Niagara and 40-processor Cray MTA-2 solving SAT equations reduced from k-clique problems in genome biology.

In Figure 6, the performance of the parallel SP code is compared between the Sun Niagara and Cray MTA-2. The parallel SP code was applied to SAT equations that were generated by the k-clique to SAT reduction algorithm. The k-clique problems were derived from the bionetwork analysis of protein homology networks.

The first problem was a 67-clique problem performed on a graph consisting of 148 nodes and 6,186 edges. This clique problem reduced to SAT equations consisting of 10,064 variables and 36,117,724 clauses. The second problem was a 3-clique problem performed on a graph consisting of 3,741 nodes and 217,350 edges. This problem reduced to SAT equations consisting of 11,223 variables and 61,979,313 clauses.

As shown by the actual clock times of Figure 6, the parallel SP algorithm implementation scales non-linearly with the size of the problem instance. Overall, the MTA-2 had a 2.28 to 5.79 times improvement in execution times over the Niagara. The speedup charts of Figure 6 also show that the parallel SP algorithm exhibits an almost linear speedup within the same problem size when utilizing from one to eight processors on the Niagara and from one to sixteen processors on the MTA-2. The efficiency generally degrades on both machines, however, after reaching those processor thresholds. Furthermore, the MTA-2 more than doubled the speedup rates of Niagara for the larger SAT problems when running on sixteen or more processors. For the bionetwork-related SAT equations, MTA-2 was able to achieve a speedup rate of up to 15.97, while Niagara's highest speedup rate was 6.33.

## Conclusion

In this paper, we introduced a high-performance computational framework for graph analysis called BioGraphE. The general approach of BioGraphE is to identify and deploy complex graph algorithms, which may be computationally- and/or data-intensive, and to integrate those algorithms with powerful and efficient computational solvers and HPC systems. The goal is to bring high-performance software and hardware capabilities to bear on challenging graph problem without requiring the scientist to become an expert of specific computing environments.

For our initial implementation, we selected a SAT solver known as Survey Propagation (SP) to serve as our first core solver. Both SAT and SP have desirable properties such as high expressivity, potential for parallelization, strong history of research, and existing tools and technologies. Our initial findings on the performance of SAT and SP were promising in that we were able to solve large k-clique problems and see definitive improvements in execution speed.

We will continue to optimize the parallel SP code to achieve the highest performance possible. We will also continue to study graph-to-SAT reductions to better understand the phase transition of generated SAT equations and how SAT solving performance maps back to the structure and properties of bionetworks. Also, we will continue to explore and experiment with other computational solvers to evolve and extend BioGraphE's overall graph analysis capabilities.

## Methods

### Serial Survey Propagation SAT solver

SP is an iterative message-passing algorithm that represents Boolean formulas as bipartite graphs, where Boolean variables correspond to one type of node and clauses to the second. Edges between the two types of nodes correspond to variables appearing in the clauses of the original formula. Messages or data passed among the nodes of the graph consists of probability distributions which are used to compute weights on the individual nodes. The SP algorithm finds the variable node that is most biased towards a true or false value and sets that node accordingly. It then updates the probability distributions, recomputes the weights, and finds and sets the next most biased node. Normally, SP will partially solve the equations to the point where a traditional SAT solver can easily complete the remaining parts of the solution. For the traditional SAT solver, we employed *Walksat *[9], which is a well-known, traditional local search algorithm.

### Parallel Survey Propagation SAT solver

Our initial analysis of the serial SP code available from Mézard & Zecchina [6] indicated that the main opportunities for parallelization in this C implementation are found in the computation of the per-variable information and the per-clause information, which can be executed in parallel. However, the overall structure of the application involves a large number of sequential iterations over the short parallel steps described above as well as several sequential steps such as choosing the most biased variables. Thus, this original structure would not ultimately be conducive to scalable performance.

We implemented a parallel version of SP from first principles based on the distributed SP algorithm presented in [11]. The main difference between the serial and parallel SP algorithms is that the decisions about which Boolean variables to fix to a particular value are done locally instead of globally. In the original serial algorithm, the variables that are fixed to particular Boolean values are selected globally after updating the weights for all variables. In the parallel version, the decision to fix a variable to a particular value is done with local information that does not depend on the state of other Boolean variables. For this reason, the distributed SP algorithm can be structured as a set of serial iterations over two large parallel steps – updating the weights of the variable nodes and updating the weights of the clause nodes. The fixed-point stopping conditions can be computed locally with respect to each variable and clause node.

### OpenMP implementation

Our OpenMP implementation reuses part of the code from the serial SP implementation to handle file input and output and most data structures, however, the main computational structure has been replaced with a new parallel structure as previously described. The computation is based on two main parallel loop nests that update the nodes representing Boolean variables and the nodes representing clauses. OpenMP threads will update a set of variables and a set of clauses using a block-dynamic scheme to reduce load imbalance. Each OpenMP thread has a separate condition flag to indicate whether a fixed-point has been reached. No node under the control of the thread changed its weight above the threshold. Special care must be taken to safely simplify the overall Boolean formula when a variable is fixed to particular value – not only the clauses in which the variable appears are affected, but other variables on the same clauses could have their values fixed as a consequence.

### Cray multithreaded implementation

Our Cray multithreaded version was derived from the OpenMP version and has basically the same computational structure. The main difference is that this version can take advantage of the memory latency hiding capabilities inherent in Cray's multithreaded processors, thus enabling very high processor utilization and scalability. However, since SP depends on a final postprocessing step executed by a traditional sequential SAT solver (*Walksat*), our experimental Cray version only implements the SP algorithm itself. Sequential code sections execute with very poor performance on the multithreaded processors.

### K-clique to SAT reduction algorithm

We implemented a simple k-clique to SAT reduction algorithm that worked on the basis of the following three simple rules:

1. Every member of the k-clique must be some node in the graph.

2. Any given node of the graph can be at most one member of the k-clique.

3. Two nodes that do not share an edge cannot both be members of the k-clique.

Each rule leads to the generation of a set of disjunctive Boolean clauses, which may be viewed as constraints on the solution. The reduction software applies these rules and generates the clauses in a form suitable for the BioGraphE SAT solver programs.

## Competing interests

The authors declare that they have no competing interests.

## Authors' contributions

GCJ, DGC, and HJS conceived and developed the BioGraphE approach. DGC implemented parallel versions of the SP code. GCN implemented visual analysis tools and k-clique to SAT reduction algorithms. GCJ and DGC conducted performance testing of algorithms and tools on HPC machines. HJS directed and managed overall research. All authors contributed to and approved the final manuscript.

## References

[B1] Cannataro M, Talia D, Srimani PK (2002). Parallel data intensive computing in scientific and commercial applications. Parallel Computing.

[B2] Altschul SF, Madden TL, Schaffer AA, Zhang J, Zhang Z, Miller W, Lipman DJ (1997). Gapped BLAST and PSI-BLAST: A new generation of protein database search programs. Nucleic Acids Research.

[B3] Oehmen CS, Nieplocha J (2006). ScalaBLAST: A scaleable implementation of BLAST for high-performance data-intensive bioinformatics analysis. IEEE Transactions on Parallel and Distributed Systems.

[B4] Campbell EA, Greenwell R, Anthony JR, Wang S, Lim L, Sofia HJ, Donohue TJ, Darst SA A conserved structural module regulates transcriptional response to diverse stress signals in eubacteria. Molecular Cell.

[B5] Sofia HJ, Nakamura GC Similarity Box: visual analytics for large sequence sets. Bioinformatics.

[B6] Mézard M, Zecchina R (2002). Random K-satisfiability problem: from an analytic solution to an efficient algorithm. Phys Rev E Stat Nonlin Soft Matter Phys.

[B7] Braunstein A, Mézard M, Zecchina R (2005). Survey propagation: An algorithm for satisfiability. Random Structures & Algorithms.

[B8] Iwama K, Miyazaki S (1994). SAT-variable complexity of hard combinatorial problems. IFIP Transactions A-Computer Science and Technology.

[B9] Selman B, Kautz H, Cohen B, Johnson DS, Trick MA (1996). Local search strategies for satisfiability testing. Cliques, Coloring, and Satisfiability: Second DIMACS Implementation Challenge.

[B10] Singer D, El-Ghazali T (2006). Parallel resolution of the Satisfiability Problem: A survey. Parallel Combinatorial Optimization.

[B11] Chavas J, Furtlehner C, Mézard M, Zecchina R (2005). Survey-propagation decimation through distributed local computations. J Stat Mech.

[B12] Anderson W, Briggs P, Hellberg CS, Hess DW, Khokhlov A, Lanzagorta M, Rosenberg R, Koelbel C, Horner-Miller B (2003). Early experience with scientific programs on the Cray MTA-2. Proceedings of the 2003 ACM/IEEE conference on Supercomputing: 15–21 November 2003; Phoenix.

[B13] Feo J, Harper D, Kahan S, Konecny P, Valero M, Ramirez A (2005). Eldorado. Proceedings of the 2nd Conference on Computing Frontiers: 4–6 May 2005; Ischia, Italy.

[B14] Nieplocha J, Marquez A, Feo J, Chavarria-Miranda D, Chin G, Scherrer C, Beagley N, Dubois M, Strenström P (2007). Evaluating the potential of multithreaded platforms for irregular scientific computations. Proceedings of the 4th Conference on Computing Frontiers: 7–9 May 2007; Ischia, Italy.

[B15] Bader DA, Feo J, Feng W, Duato J (2005). On the architectural requirements for efficient execution of graph algorithms. Proceedings of the 2005 International Conference on Parallel Processing: 14–17 June 2005; Oslo, Norway.

[B16] Bader DA, Madduri K, Feng W (2006). Designing multithreaded algorithms for breadth-first search and st-connectivity of the Cray MTA-2. Proceedings of the 2006 International Conference on Parallel Processing: 14–18 August 2006; Columbus, Ohio.

